# Long non-coding RNA RHPN1-AS1 promotes tumorigenesis and metastasis of ovarian cancer by acting as a ceRNA against miR-596 and upregulating LETM1

**DOI:** 10.18632/aging.102911

**Published:** 2020-03-12

**Authors:** Junrong Wang, Weimin Ding, Yingke Xu, Enfu Tao, Miaojun Mo, Wei Xu, Xu Cai, Xiaomin Chen, Junhui Yuan, Xiuying Wu

**Affiliations:** 1 Department of Laboratory Medicine, Wenling Maternal and Child Health Care Hospital, Wenling 317500, Zhejiang Province, China; 2 Department of Obstetrics and Gynecology, Wenling Maternal and Child Health Care Hospital, Wenling 317500, Zhejiang Province, China; 3 Department of Pediatrics, Wenling Maternal and Child Health Care Hospital, Wenling 317500, Zhejiang Province, China; 4 Department of Obstetrics and Gynecology, Women’s Hospital School of Medicine Zhejiang University, Hangzhou 310000, Zhejiang Province, China

**Keywords:** epithelial ovarian cancer (EOC), RNA stability, m6A modification, RHPN1-AS1, miR-596

## Abstract

Background: In recent decades, long non-coding RNAs (lncRNAs) have been reported as crucial functional regulators involved in ovarian cancer. In the present study, we explored how lncRNA RHPN1-AS1 influences the progression of epithelial ovarian cancer (EOC) through tumor cell-dependent mechanisms.

Results: The expression of RHPN1-AS1 in EOC tissues was higher than that in para-cancerous control tissues. High expression of RHPN1-AS1 was closely associated with poor prognosis in EOC patients. N6-methyladenosine (m6A) improved the stability of RHPN1-AS1 methylation transcript by reducing RNA degradation, which resulted in upregulation of RHPN1-AS1 in EOC. In vitro and in vivo functional experiments showed that RHPN1-AS1 promoted EOC cell proliferation and metastasis. RHPN1-AS1 acted as a ceRNA to sponge miR-596, consequently increasing LETM1 expression and activating the FAK/PI3K/Akt signaling pathway.

Conclusion: RHPN1-AS1-miR-596-LETM1 axis plays a crucial role in EOC progression. Our findings may provide promising drug targets for EOC treatment.

Methods: We determined the aberrantly expressed lncRNAs in EOC via microarray analysis and validated RHPN1-AS1 expression by qRT-PCR. The RHPN1-AS1-miR-596-LETM1 axis was examined by dual-luciferase reporter assay and RIP assay. The mechanism of RHPN1-AS1 was investigated through gain- and loss-of-function studies both in vivo and in vitro.

## INTRODUCTION

Epithelial ovarian cancer (EOC) has been reported to be the most lethal gynecologic malignancy worldwide [1]. Although standard treatments, including primary deburring surgery (PDS), platinum/taxane-based chemotherapy, and neoadjuvant chemotherapy have dramatically ameliorated the overall survival (OS) of EOC patients, the 5-year OS remains very low [2]. High throughput sequencing technologies made it possible for large-scale access to gene expression profiles. Gene expression microarray provided new insights for the identification of tumor-associated genes, biomarkers, and therapeutic targets [3]. The integration of those databases containing multiple gene expression data of various cancer types allows in-depth analysis of molecular mechanisms.

Long non-coding RNAs (lncRNAs) have been widely engaged in various cellular processes, including post-transcriptional regulation via epigenetic regulation [4, 5]. In addition, growing evidence has shown that lncRNAs function critically in multiple diseases, especially in cancer occurrence and progression [6, 7]. Recently, lncRNAs, such as FLVCR1-AS1, ABHD11-AS1, and NORAD have been found to be associated with tumorigenesis, metastasis, and progression of EOC and are regarded as potential therapeutic targets for EOC [8–10]. It has been reported that dysregulation of lncRNAs played essential roles in the development of tumors, including EOC [11]. LncRNA RHPN1-AS1 is an important regulator in cancer development and progression. It has been reported that RHPN1-AS1 enhances cell proliferation, migration, and in cervical cancer via miR-299-3p/FGF2 cascade [12]. RHPN1-AS1 regulates breast cancer cell proliferation via miR-4261/c-Myc axis and modulation of p53 [13]. RHPN1-AS1 also functions in glioma, neck squamous cell carcinoma, gastric cancer, hepatocellular carcinoma, non-small cell lung cancer, and Uveal Melanoma [14–19]. However, the function of RHPN1-AS1 in tumorigenesis of EOC, are still largely unknown. Therefore, further understanding of RHPN1-AS1 regulation mechanisms will provide useful biomarkers and individualized therapies for the prognosis of EOC.

MiRNAs are a group of small non-coding RNAs, whose aberrant expression is highly associated with many diseases, including cancers [20–22]. It has been shown that overexpression of miR-596 suppresses MAPK/ERK signaling and thus inhibits melanoma cell survival, migration, and invasion [20]. MiR-596 has also been reported to enhance the sensitivity of osteosarcoma cells to anlotinib by repressing Survivin expression [21]. But the potential function of miR-596 in ECO has not been well-characterized.

Leucine zipper/EF hand-containing transmembrane-1 (LETM1) is a mitochondrial inner membrane protein that is a potential biomarker of prognosis of several cancers, such as colorectal cancer, esophageal squamous cell carcinoma, breast cancer, lung non-small cell carcinoma, and gastric adenocarcinoma [23–27]. It has been demonstrated that knockdown of LETM1 suppresses the proliferation and invasion of bladder cancer cells [28], indicating an oncogenic role of LETM1 in cancer. However, the relationship between LETM1 and RHPN1-AS1 and whether LETM1 has a critical role in EOC progression remain unknown.

In the current study, we aimed to identify whether lncRNA RHPN1-AS1 has a significance critical function in EOC. Our study elucidated the regulatory mechanism of RHPN1-AS1 in EOC via modulation of miR-596/LETM1, which may provide a prognostic indicator and promising therapeutic target for EOC patients.

## RESULTS

### Clinical significance of RHPN1-AS1 in EOC

**Figure 1:**
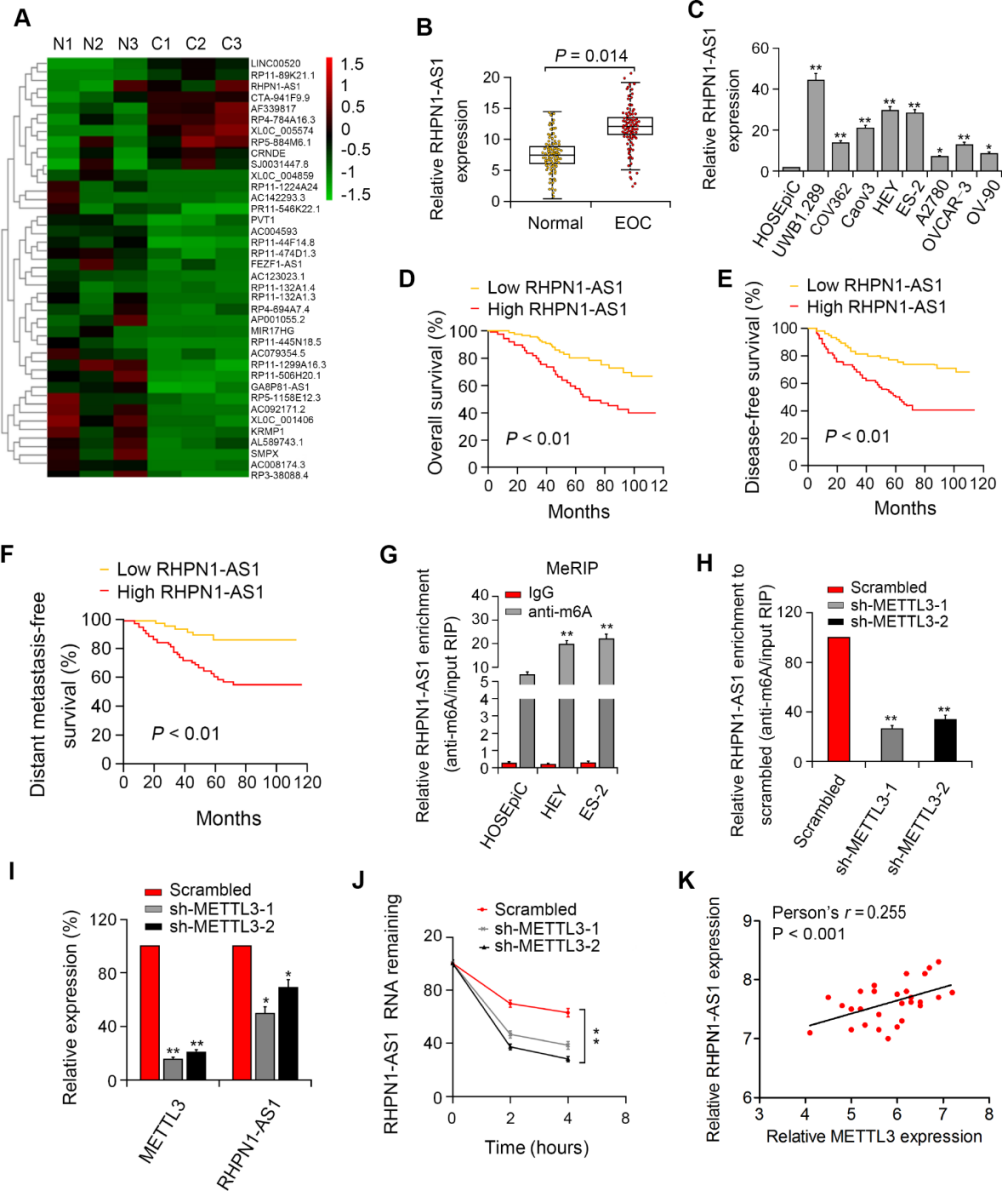
**RHPN1-AS1 is overexpressed in EOC tissues and cells and predicts poor prognosis.** (**A**) The heat map of lncRNAs with the largest difference between the three pairs of EOC and control tissues (P < 0.05). (**B**, **C**) RHPN1-AS1 expression in EOC specimens, control tissues, and EOC cell lines. (**D**–**F**) Association between RHPN1-AS1 expression and survival by Kaplan–Meier analysis. (**G**) m6A methylation level of RHPN1-AS1 in EOC cells and HOSEPIC cells determined by MeRIP-qPCR. (**H**) Changes in m6A-modified RHPN1-AS1 levels after METTL3 sectioning in HEY cells. (**I**) METTL3 and RHPN1-AS1 expressions in sh-METTL3-HEY cells. (**J**) RHPN1-AS1 stability was decreased in HEY cells with METTL3 knockdown. (**K**) Correlation between RHPN1-AS1 and METTL3 expression in EOC tissues.*P<0.05, **P<0.01.

Microarray analysis was performed with three EOC species and para-cancerous control tissues. We identified 326 dysregulated lncRNAs (folding changes ≥ 2, P < 0.05), of which RHPN1-AS1 was one of the most upregulated lncRNAs in EOC (Figure 1A). Besides, the expression of RHPN1-AS1 in 86 EOC tissues and para-cancerous control tissues was examined by qRT-PCR. We found that RHPN1-AS1 was significantly upregulated in EOC tissue samples compared to control tissues (Figure 1B, P=0.014). Similarly, the expression of RHPN1-AS1 in 9 EOC cells was significantly higher than that in human ovarian epithelial cells (HOSEpiC) (Figure 1C, P < 0.01). Second, all EOC patients were divided into two groups: high/low RHPN1-AS1 expression groups by a median cutoff value. We found that high RHPN1-AS1 level was significantly associated with distant metastasis and death (P < 0.05; Supplementary Table 1). Additionally, Kaplan-Meier survival analysis demonstrated that patients with high RHPN1-AS1 expression had worse OS and DFS (Figure 1D–1F, P < 0.01). It is suggested that RHPN1-AS1 expression is an important indicator to judge the prognosis of EOC.

### m6A modification enriches in RHPN1-AS1 and enhances its transcriptional stability

To investigate the presence of m6A modification in RHPN1-AS1, we first predicted the m6A locus using the bioinformatics pipeline M6avar (http://m6avar.renlab.org/) and identified the M6A sequence motifs in the last exon (positions 1794 and 3528). Next, we performed RIP analysis on HEY, UWB1.289, and HOSEpiC cells. The level of m6A in UWB1.289 and HEY cells was significantly higher than that in HOSEpiC cells (Figure 1G, P < 0.01). Then, shRNA-METTL3 was transfected into HEY cells, and it was found that knockdown of METTL3 resulted in reduced levels of m6 of total RNA and RHPN1-AS1 (Figure 1H, P < 0.01). We then investigated whether m6A modification would affect RNA metabolism of RHPN1-AS1. Knockdown of METTL3 remarkably decreased the expression of RHPN1-AS1 (Figure 1I, P < 0.01). After blocking new RNA synthesis with actinomycin D, we measured the loss of RHPN1-AS1. The results showed that the stability of RHPN1-AS1 was reduced after METTL3 silencing (Figure 1J, P < 0.01). Moreover, RHPN1-AS1 is positively associated with METTL3 in EOC tissues (Figure 1K, P < 0.001). These findings demonstrated that the m6A level of RHPN1-AS1 was elevated in EOC, and the m6A modification of RHPN1-AS1 increased its transcriptional stability, which may be part of the reason that RHPN1-AS1 was considerably upregulated in EOC.

### RHPN1-AS1 promotes EOC cell viability and mobility

**Figure 2:**
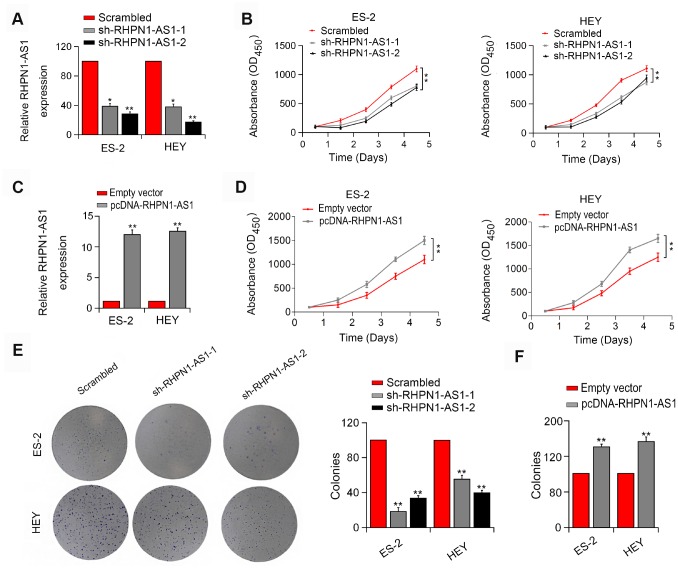
**RHPN1-AS1 enhances ES-2 and HEY cell proliferation and colony formation.** (**A**, **C**) RHPN1-AS1 expression in ES-2 and HEY cells transfected with sh-RHPN1-AS1s or pcDNA- RHPN1-AS1. (**B**, **D**) CCK-8 assay in ES-2 and HEY cells transfected with sh-RHPN1-AS1s or pcDNA-RHPN1-AS1 by. (**E**, **F**) Colony formation assay in ES-2 and HEY cells transfected with sh-RHPN1-AS1s or pcDNA-RHPN1-AS1. *P<0.05, **P<0.01.

**Figure 3:**
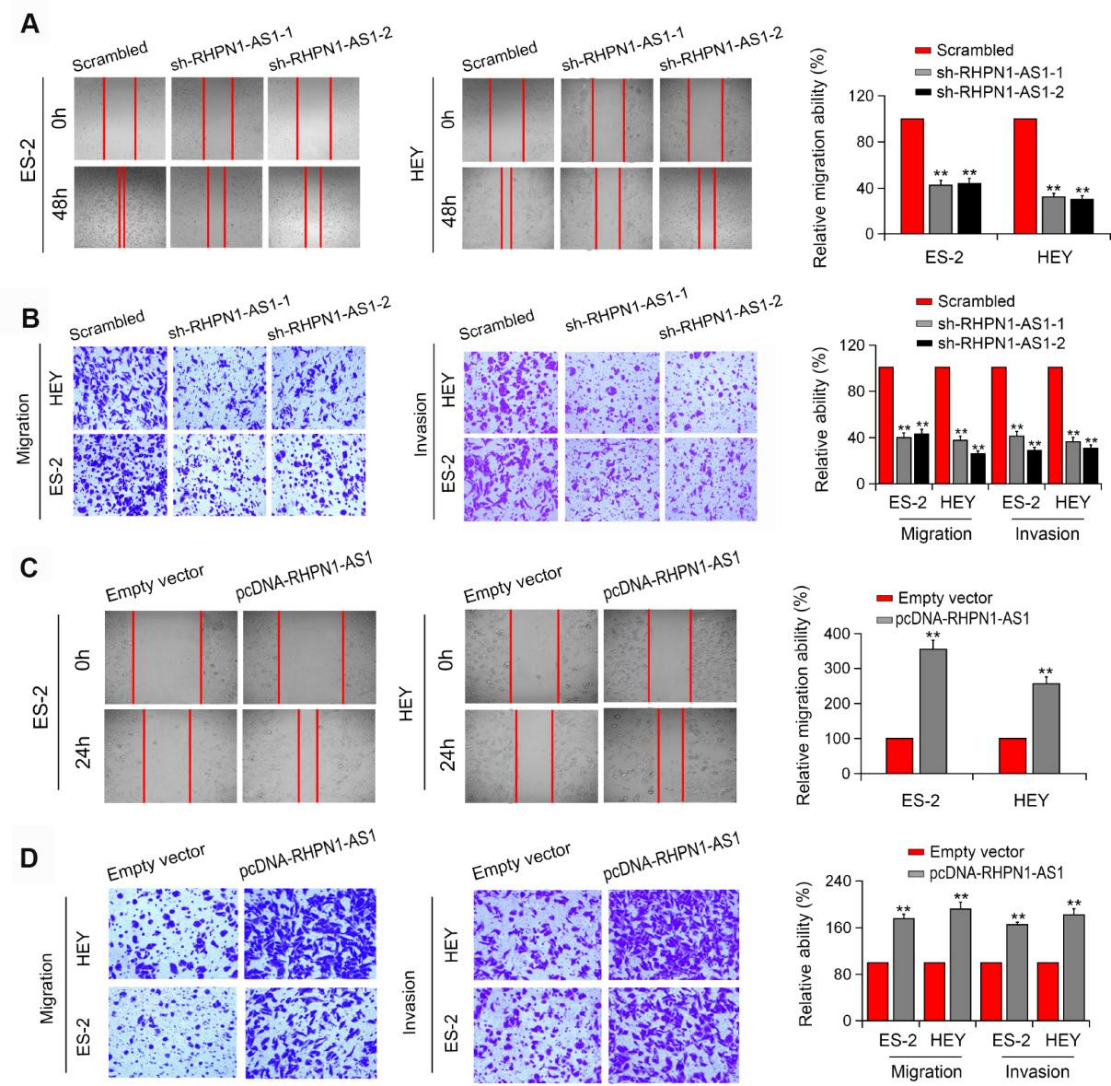
**RHPN1-AS1 enhances EOC cell migration and invasion.** (**A**, **B**) Wound healing assay and Transwell assay of ES-2 and HEY cells transfected with sh-RHPN1-AS1s. (**C**, **D**) Wound healing assay and Transwell assay of ES-2 and HEY cells transfected with pcDNA-RHPN1-AS1. *P<0.05, **P<0.01.

To assess the effect of RHPN1-AS1 on EOC cell viability and mobility, HEY and ES-2 EOC cells were transfected with RHPN1-AS1 shRNAs (sh-RHPN1-AS1-1 and -2). The knockdown efficiencies of RHPN1-AS1 shRNAs in HEY and ES-2 cells were confirmed by quantitative RT-PCR (Figure 2A). CCK-8 and colony formation analysis showed that the viability and clonogenic ability of HEY and ES-2 cells were significantly inhibited in the sh-RHPN1-AS1-transfected groups (Figure 2B, 2C). In contrast, overexpression of RHPN1-AS1 enhanced the growth and colony formation of EOC cells (Figure 2C, 2D, 2F). Moreover, wound healing assay and transwell assays further demonstrated that RHPN1-AS1 depletion repressed ES-2 and HEY cell migration and invasion (Figure 3A, 3B, P < 0.01). On the contrary, RHPN1-AS1 overexpression enhanced the migration and invasion of ES-2 and HEY cells (Figure 3C, 3D, P < 0.01). Taken together, these results suggested that RHPN1-AS1 acts as a carcinogenic lncRNA in EOC.

**Figure 4:**
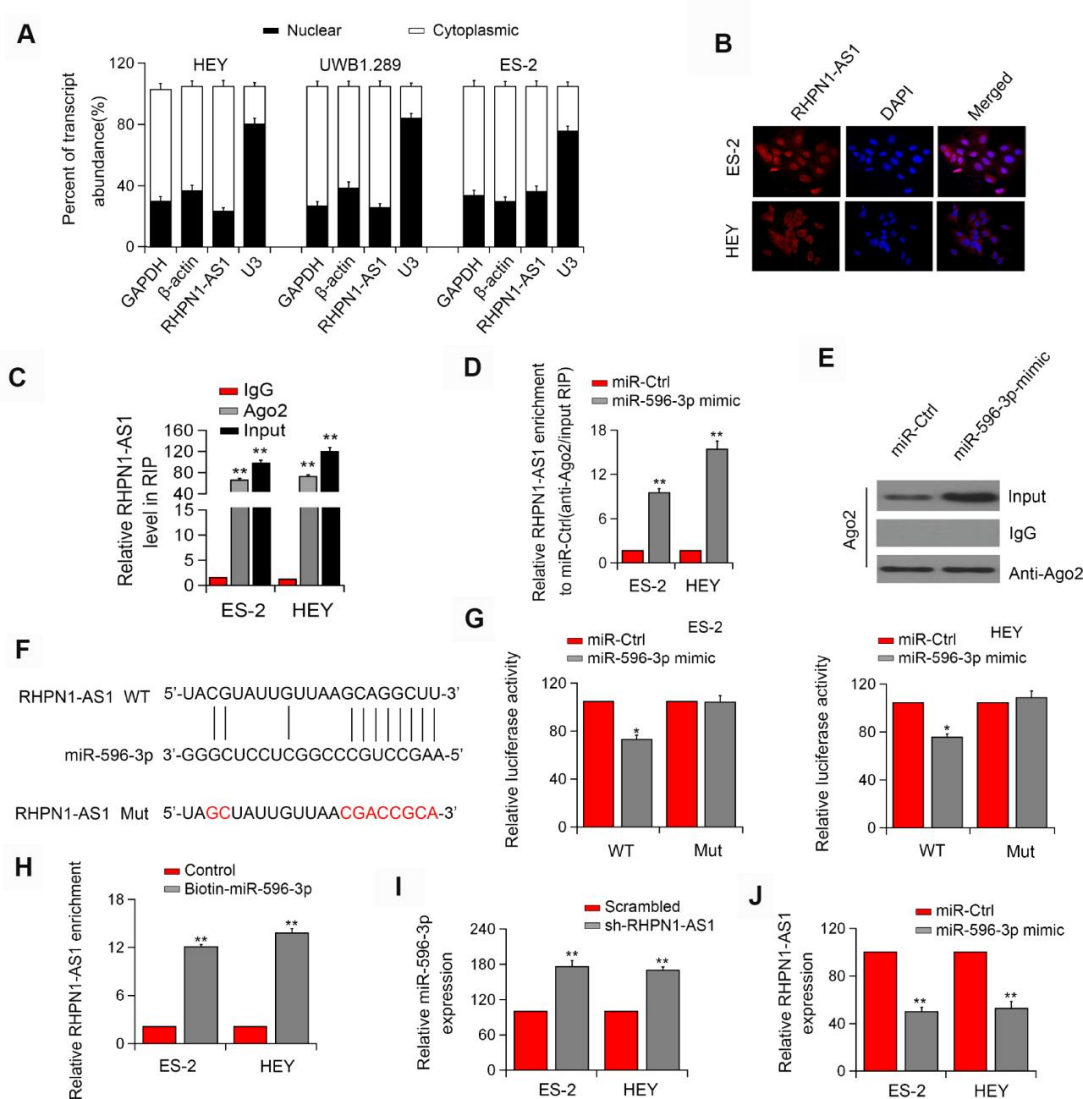
**RHPN1-AS1 acts as a ceRNA to sponge miR-596.** (**A**) qRT-PCR analysis of RHPN1-AS1 expression in HEY, UWB 1.289, and ES-2 cells. (**B**) RNA-FISH showed the subcellular localization of RHPN1-AS1 in ES-2 and HEY cells. RHPN1-AS1 was stained with Cy3 (red) and nuclei were stained with DAPI (blue). (**C**) Ago2-RIP was performed in ES-2 and HEY cells. (**D**, **E**) Enrichment of RHPN1-AS1 in ES-2 and HEY cells transfected with miR-596 mimic. (**F**) Prediction of miR-596 binding site in the RHPN1-AS1. (**G**) Luciferase activity in ES-2 and HEY cells co-transfected with WT/mut RHPN1-AS1 plasmids, and miR-596 mimic or miR-Ctrl. (**H**) Biotin-miR-596 pull-down enrichment of RHPN1-AS1. (**I**) Relative levels of miR-596 in ES-2 and HEY cells transfected with sh-RHPN1-AS1#2 or scrambled control. (**J**) Relative levels of RHPN1-AS1 in ES-2 and HEY cells transfected with miR-596-mimic or miR-Ctrl. *P<0.05, **P<0.01.

### RHPN1-AS1 acts as a ceRNA and binds to miR-596

Next, we determined the localization of RHPN1-AS1 in cells and found that RHPN1-AS1 was primarily localized in the cytoplasm of HEY, ES-2, and UWB1.289 cell lines (Figure 4A, P < 0.05). Similar result was obtained by the FISH assay (Figure 4B). The endogenous RHPN1-AS1 was enriched in Ago2-RIP (Figure 4C, P < 0.01), indicating that RHPN1-AS1 might act as a ceRNA. Ago2-RIP analysis also showed that the content of RHPN1-AS1 in the miR-596 overexpression group was significantly higher than that in the miR-Ctrl group (Figure 4D–4E, P < 0.05). Dual-Luciferase reporter analysis showed that overexpression of miR-596 reduced the luciferase activity of RHPN1-AS1-WT but had no significant effect on RHPN1-AS1-Mut (Figure 4F–4G, P < 0.05). Besides, the biotin-labeled miRNA pull-down assay showed that the expression of RHPN1-AS1 was increased in EOC cells transfected with biotin-labeled miR-596 in comparison to the control group (Figure 4H, P < 0.05). Moreover, RHPN1-AS1 silencing significantly enhanced the expression of miR-596 (Figure 4I, P < 0.01). Meanwhile, miR-596 overexpression markedly inhibited the level of RHPN1-AS1 (Figure 4J, P < 0.01).

### RHPN1-AS1 upregulates LETM1 by sponging miR-596

**Figure 5:**
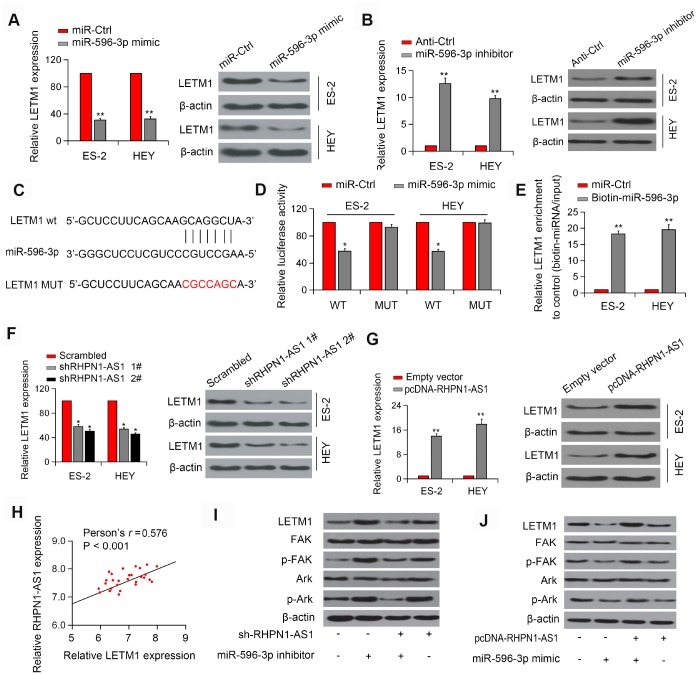
**RHPN1-AS1 acts as a sponge for miR-596 to increase LETM1 expression.** (**A**, **B**) Relative mRNA and protein levels of LETM1 in ES-2 and HEY cells transfected with miR-596-mimics or miR-596 inhibitor. (**C**) The predicted binding site of miR-596 in the LETM13'-UTR. (**D**) Luciferase activity in ES-2 and HEY cells co-transfected with WT/mutant LETM1 plasmids with miR-596 mimic or miR-Ctrl. (**E**) Enrichment of LETM1 by biotin-miR-596. (**F**, **G**) LETM1 protein levels in ES-2 and HEY cells transfected with sh-RHPN1-AS1s or pcDNA-RHPN1-AS1. (**H**) Correlation between RHPN1-AS1 and LETM1 expression in EOC tissues. (**I**) Expression levels of LETM1, FAK, p-FAK, and AKT, and p-Akt in cells transfected with shRHPN1-AS1#2 and/or miR-596 inhibitor. (**J**) Expression levels of LETM1, FAK, p-FAK, AKT, and p-Akt in cells transfected with pcDNA-RHPN1-AS1 and/or miR-596 mimic. *P<0.05, **P<0.01.

Next, TargetScan and miRanda were applied to predict potential target genes for miR-596. Interestingly, the expression of LETM1 could be modulated by miR-596 and thus was selected for further study (Figure 5A–5B, P < 0.01). Luciferase reporter assay showed that overexpression of miR-596 inhibited the luciferase activity of LETM1-WT but did not significantly alter the activity of LETM1-Mut in EOC cells (Figure 5C, 5D). In addition, pull-down analysis confirmed that LETM1 interacted with miR-596 (Figure 5E, P < 0.01). Additionally, knockdown of RHPN1-AS1 decreased LETM1 expression, while overexpression of RHPN1-AS1 had a positive effect (Figure 5F–5G, P<0.01). We also detected a positive correlation between RHPN1-AS1 and LETM1 expression in EOC tissues (Figure 5H, r=0.576, P < 0.001). These results indicated the presence of the RHPN1-AS1/miR-596/LETM1 regulatory axis. Next, we investigated the regulatory role of RHPN1-AS1 in the PI3K/Akt signaling pathway. RHPN1-AS1 silencing reduced the expressions of LETM1, p-FAK and p-Akt, which were reversed by transfection with miR-596 inhibitor (Figure 5I). On the contrary, RHPN1-AS1 overexpression elevated the levels of LETM1, p-FAK and p-Akt, which were reversed after miR-596-mimic transfection (Figure 5J). Together, these findings indicated that RHPN1-AS1 sponged miR-596 to upregulate LETM1 and activate the FAK/PI3K/Akt pathway.

### LETM1 overexpression inhibits EOC cell growth

**Figure 6:**
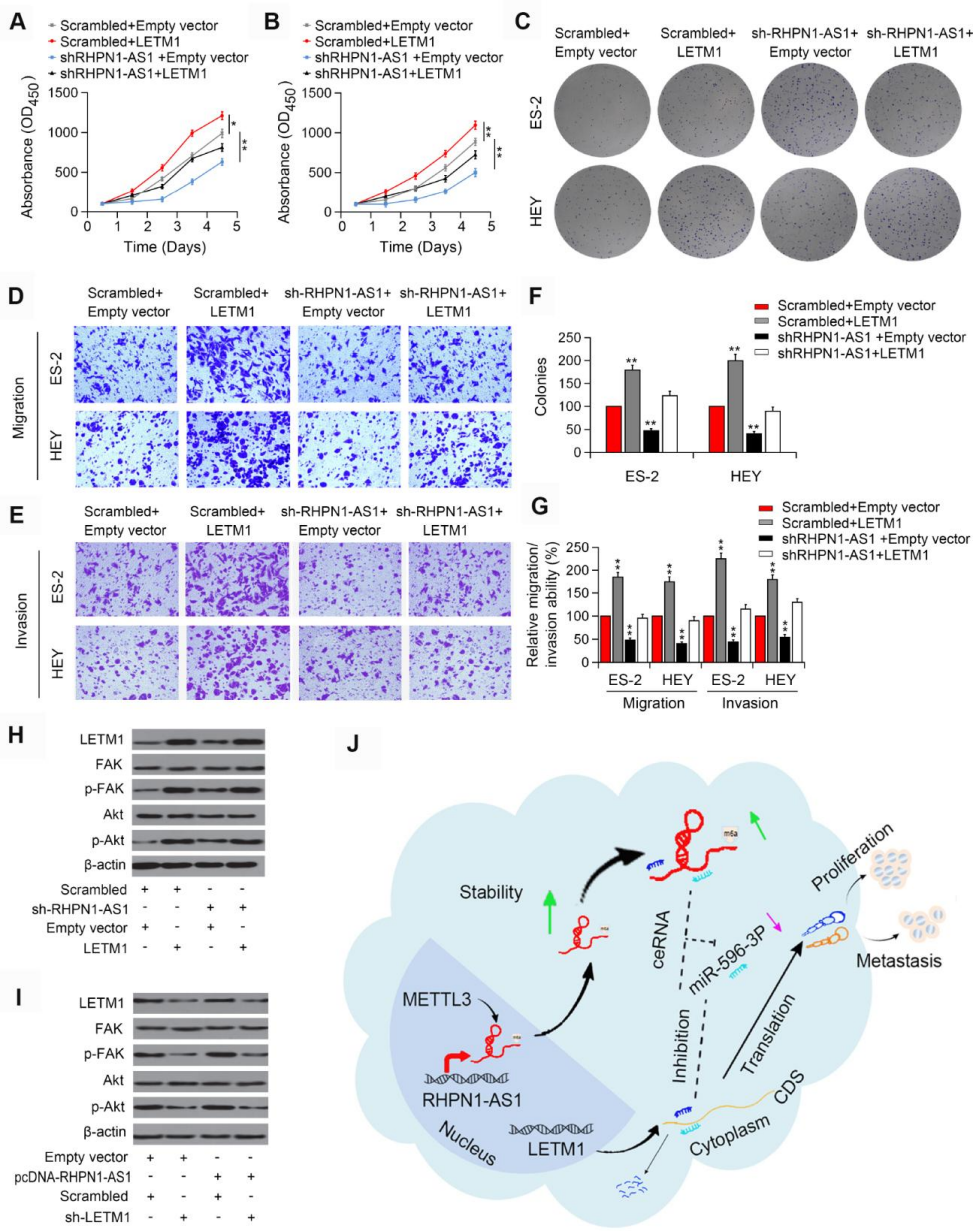
**LETM1 is involved in RHPN1-AS1-mediated EOC progression.** (**A**, **B**) CCK-8 assay in ES-2 and HEY cells transfected with shRHPN1-AS1#2 and/or pcDNA-LETM1. (**C**) Colony formation analysis in EOC cells transfected with shRHPN1-AS1#2 and/or pcDNA-LETM1. (**D**–**G**) Transwell assay in EOC cells transfected with shRHPN1-AS1#2 and/or pcDNA-LETM1. (**H**) Western blotting analysis of LETM1, FAK, p-FAK, Akt, p-Akt in EOC cells transfected with shRHPN1-AS1#2 and/or pcDNA-LETM1. (**I**) Western blotting analysis of LETM1, p-FAK, Akt, p-Akt in ES-2 and SUNE1 cells transfected with pcDNA-RHPN1-AS1 and/or sh-LETM1. (**J**) A mechanism model for the expression and function of RHPN1-AS1 in EOC. *P<0.05, **P<0.01.

To demonstrate that RHPN1-AS1 promotes tumor progression in a LETM1-dependent manner, we transfected ES-2 and HEY cells stably overexpressing sh-RHPN1-AS1 with LETM1 overexpression vector. In ES-2 and HEY cells, in vitro functional experiments showed that recovery of LETM1 partially rescued the viability and mobility of EOC cells inhibited by RHPN1-AS1 knockdown (Figure 6A–6G, P < 0.01). Besides, overexpression of LETM1 increased the phosphorylation levels of FAK and Akt that were inhibited by RHPN1-AS1 downregulation (Figure 6H). On the contrary, depletion of LETM1 abolished the enhancive effects of RHPN1-AS1 on the phosphorylation of FAK and Akt (Figure 6I). These findings indicated that RHPN1-AS1 is a carcinogenic lncRNA that promotes EOC cell growth and metastasis via the PI3K/Akt signaling pathway (Figure 6J).

### RHPN1-AS1 promotes tumorigenesis in EOC cells in vivo

**Figure 7:**
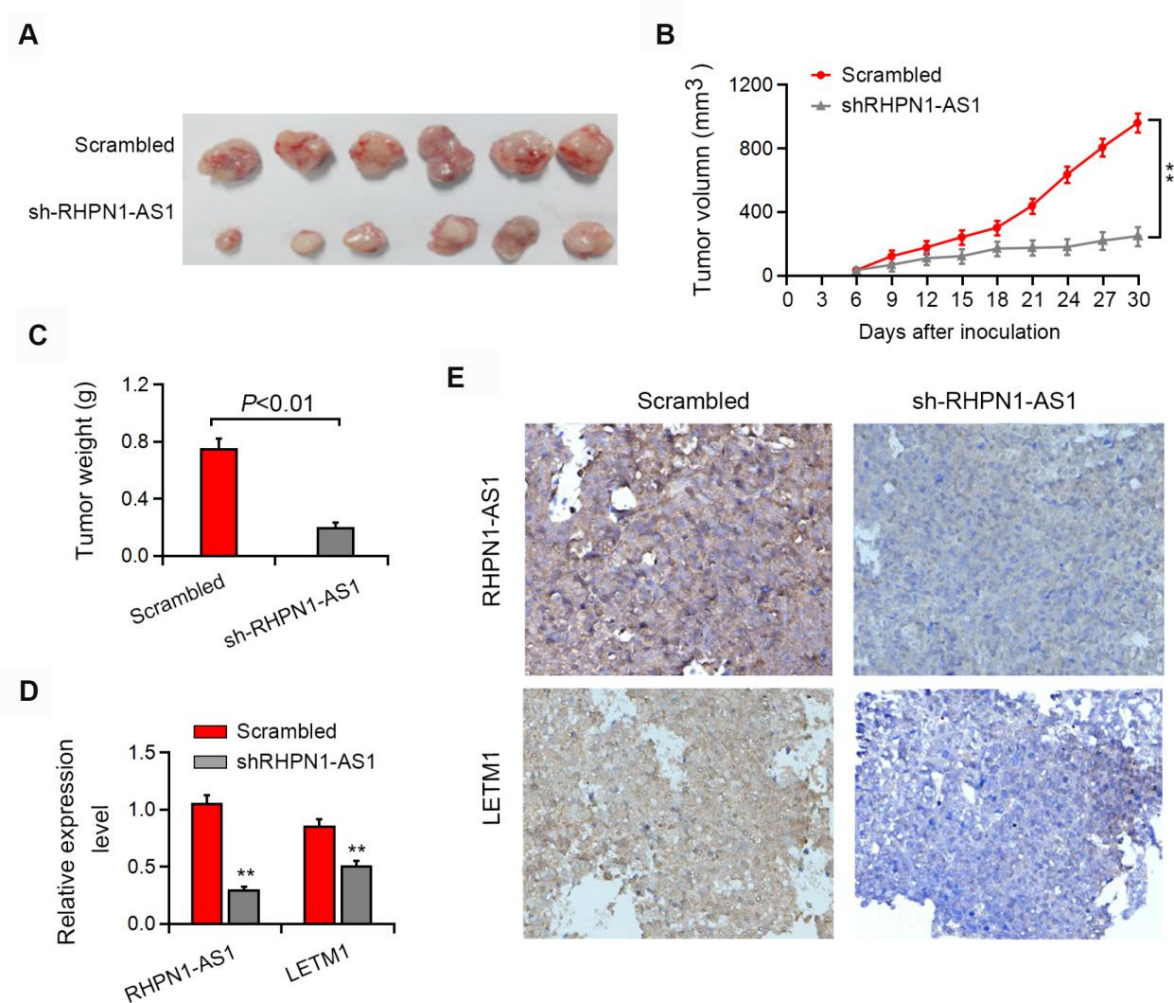
**RHPN1-AS1 enhances EOC tumorigenesis in vivo.** (**A**–**C**) Representative images of the formed tumor, tumor volume and tumor weight. (**D**–**E**) qRT-PCR, in situ hybridization and immunohistochemistry were used to detect the expression of RHPN1-AS1 and LETM1. *P<0.05,**P<0.01.

Next, we tested whether RHPN1-AS1 has the tumor-forming ability in vivo. Xenograft tumor model showed that knockdown of RHPN1-AS1 significantly reduced the subcutaneous tumor growth of EOC cells (Figure 7A–7C, P < 0.05). Moreover, LETM1 expression was significantly downregulated in tumors formed from RHPN1-AS1 knockdown cells compared with control cells (Figure 7D, 7E). We further found that restoration of LETM1 expression partially rescued the inhibition of tumor growth by RHPN1-AS1 silencing (data not shown).

## DISCUSSION

There is increasing evidence that dysregulation of lncRNAs participates in the development and progression of different cancers [29]. Here, we found that lncRNA RHPN1-AS1 is significantly upregulated and is associated with poor survival in patients with EOC. RHPN1-AS1 acts as a ceRNA to, increase the expression of LETM1, thereby promoting the growth and mobility of EOC cells. It is suggested that RHPN1-AS1 plays a carcinogenic role in the occurrence and development of EOC and can be used as a prognostic indicator for EOC patients.

Clinically, current anatomical-based TNM staging systems do not accurately differentiate the risk of recurrence or distant metastasis in EOC patients. Therefore, substantial differences in clinical outcomes in EOC patients require new prognostic biomarkers. New evidence suggests that lncRNAs may serve as new prognostic indicators. In this study, the expression level of RHPN1-AS1 in paraffin-embedded EOC tissue samples was examined by qRT-PCR for risk stratification and prognosis prediction. Although nucleic acids were extensively degraded in paraffin-embedded tissues, they were still valuable assets for clinical transformation studies. Therefore, we used primers to generate short RHPN1-AS1 amplicons (about 60 bp) to enhance the quantitation efficiency and reliability, as previously described. Survival analysis showed that the clinical prognosis of patients with high expression of RHPN1-AS1 was reduced, suggesting that RHPN1-AS1 may be a promising prognostic biomarker to guide the development of personalized therapy in EOC patients.

In recent years, cutting-edge studies have shown that m6A has a wide range of modifications to mRNA and lncRNA, functionally regulating the eukaryotic transcriptome, affecting RNA splicing, export, localization, translation, and stability [30, 31]. The m6A modification is the most abundant internal transcript modification in eukaryotic mRNAs, introduced by the m6A-methyltransferase complex, named “writer” [32]. The factors explaining the specific modifications were identified as “readers” such as YTHDF1/2/3 and YTHDC1/2 [33]. m6A in RNA can be removed by RNA demethylase, including FTO and ALKBH5 [34]. Here, we found that m6A was abundant in RHPN1-AS1 in EOC cells. Modification of m6A in RHPN1-AS1 led to an increase in its RNA stability, which may be partially contributed to the up-regulation of RHPN1-AS1 in EOC. There may be other mechanisms, such as DNA methylation, histone modifications, and miRNA dysregulation, which deserve further exploration.

A growing body of evidence supports the existence of a new and extensive network of interactions involving CeRNAs, in which LncRNAs regulate miRNAs by competitively binding protein-encoding mRNA molecules at their target sites. For example, LINC01234 acts as a ceRNA that regulates the expression of CBFB by sponging miR-204-5p [35]. DANCR acts as a ceRNA to promote REOCK1 by acting as bait for miR-335-5p and miR-1972 in osteosarcoma [36]. UICLM regulates the expression of miRNA-215 by acting as a ceRNA, thereby promoting the metastasis of colorectal cancer [37]. Here, we found that RHPN1-AS1 was mainly located in the cytoplasm of EOC cells and acted as a sponge for miR-596.

MiR-596 contains chromosomal deletions with putative TSGs that can be downregulated in various types of cancer [38]. Our findings revealed the significance of the interaction between RHPN1-AS1 and miR-596 in tumorigenesis. RHPN1-AS1 promoted the proliferation and metastasis of EOC cells, and overexpression of miR-596 partially reversed these effects.

Using an online database, we predicted LETM1 as a potential target for miR-596 and confirmed this by luciferase reporter assay and biotinylated miRNA pull-down analysis. Furthermore, miR-596 overexpression repressed the expression of LETM1. In the present study, we demonstrated that RHPN1-AS1 bound to miR-596, which led to upregulation of LETM1 and activation of the FAK/PI3K/AKT signaling pathway, and thus promoted the viability and mobility of EOC cells.

In summary, RHPN1-AS1 promotes the proliferation, migration, and invasion of EOC cells, suggesting that RHPN1-AS1 is a carcinogenic lncRNA in EOC. RHPN1-AS1 exerts its function by acting as a ceRNA of miR-596, consequently increasing LETM1 expression and activating the FAK/PI3K/Akt pathway. Our study indicates that RHPN1-AS1 plays a vital role in the development and progression of EOC and emphasizes its importance as a prognostic indicator and therapeutic target for EOC.

## MATERIALS AND METHODS

### Clinical specimens

A total of 86 fresh frozen EOC and para-cancerous ovarian tissue samples were collected from May 2013 to Dec 2014 in Wenling Maternal and Child Health Care Hospital. All participants received no anti-cancer treatments before operation. This study was approved by the Wenling Maternal and Child Health Care Hospital Ethics Review Board and received written informed consent from all patients.

### Cell culture

EOC cell lines Caov3, ES-2, A2780, OV-90, OVCAR-3 were cultured in RPMI-1640 medium (Invitrogen, Carlsbad, CA, USA) supplemented with 10% fetal bovine serum (FBS; ScienCell, USA). EOC cell lines A2780, UWB1.289, HEY, COV362 and human ovarian epithelial cell line (HOSEpiC) cell line was cultured in DMEM (Corning, USA), supplemented with 10% FBS (ScienCell) with 5% CO_2_ and 95% humidity at 37°C.

### Microarray analysis

LncRNA expression was analyzed using the microarray (Human LncRNA Array v4.0, Arraystar, Inc). The GeneSpring GX v11.5.1 software package (Agilent Technologies, Santa Clara, Calif., USA) was used for data normalization and subsequent processing. LncRNAs aberrantly expressed were identified by stringent screening criteria (fold change ≥ 2, P < 0.05). R (version 3.3.3) software was used to draw the heat map. Microarray data have been deposited in the National Biotechnology Information Center's Gene Expression Integrated Database (accession number GSE82059).

### Real-time RT-PCR

Total RNA was extracted from cell lines or tissues using TRIzol reagent (Invitrogen, Carlsbad, CA, USA). RNA was reverse-transcribed to cDNA using a RevertAid™ First Strand DNA Synthesis Kit (Thermo Fisher Scientific, Waltham, MA, USA). Platinum SYBR-Green PCR kit (Roche Diagnostics, Indianapolis, IN, USA) was subjected to quantitative RT-PCR analysis using the ABI Q6 detection system (Applied Biosystems, Foster City, USA). Normalization was performed with GAPDH or U6 as reference genes. The relative expressions were analyzed using the 2^−ΔΔCt^ method.

### Western blot analysis

Cell lysates were separated by electrophoresis using SDS-polyacrylamide gel (4%-10%) and then transferred to a PVDF membrane (Microwell). The membrane was blocked with 5% skim milk and incubated overnight at 4°C with the following primary antibodies: anti-METTL3 (1:800; Abcam), anti-LETM1 (1:500; Abcam), anti-FAK (1:800; CST), anti-p-FAK (1:800; CST), anti-Akt (1:800; CST), anti-p-Akt (Ser-473; 1:500; CST) or anti-β-actin (1:2000; protection). The membrane was then incubated with secondary antibodies conjugated with HPR for 1 h at room temperature, and protein was detected with BeyoECLPlus (Beyotime, China).

### m6A RNA methylation quantification

The total level of extracted m6A of RNA was measured using the EpiQuik m6A RNA Methylation Quantitation Kit (colorimetric method; Epigentek, New York, USA), according to the manufacturer's instructions.

### RNA immunoprecipitation (RIP)

The EZ-Magna-RIP kit (Millipore, Billerica, USA) was used for RNA immunoprecipitation (RIP) according to the manufacturer's instructions. Briefly, 24 hours after transfection, cells were harvested using m6A antibody (2 μg/sample; Synaptic Systems, Goettingen, German) or Ago2 antibody (5 μg/sample; Abcam, Cambridge, UK) for RIP experiments. IgG was used as a negative control. Coprecipitation of RNA was detected by quantitative RT-PCR, as described above. In the analysis of RIPed RNA, a comparative Ct (ΔΔCt) method was employed. The input fraction Ct values were used to correct for differences in preparation of each set of RNA samples, and the negative control (IgG) Ct was used to adjust the background score. Primers for measuring m6A modified RHPN1-AS1 levels or for detecting RHPN1-AS1 involved in RNA-induced silencing complex (RISC) are provided in Supplementary Table 1.

### Transient transfection

shRNAs against RHPN1-AS1 or integrin β3 (LETM1) were synthesized by GENEWIZ (Suzhou, China) and cloned into the lentivirus plasmid pcDNA-EF2-puromycin (Addgene, Cambridge, USA). The pENTER-LETM1 plasmid and pENTER vector were purchased from Vigene Biosciences (Jinan, China). miR-596-3p mimic, inhibitor, as well as the negative control were purchased from Ribobio (China). Cells were transfected with liposome 3000 reagent (Invitrogen) or RNAiMAX reagent (Invitrogen). Cells were taken for further detection 48 h after transfection. shRNA-RHPN1-AS1 2# or control scrambled shRNA was inserted into pLKO.1 vector and 293T cells (PEI) (Polysciences) were co-transfected with psPAX2 packaging plasmid (Addgene) and pCMV-VSV-G envelope plasmid (Addgene, Warrington, PA, USA). After transfection, cell supernatants were used to infect HEY and ES-2 cells, and stably transfected cells were screened with puromycin (1 μg/ml) and verified by quantitative RT-PCR. The lentiviral plasmid overexpressing LETM1 (Genecooeia, Guangzhou, China) was further transfected into stable cells to obtain SUNE1 cells stably expressing shRNA-RHPN1-AS1 2# and LETM1.

### Luciferase reporter assay

The wild type or mutant RHPN1-AS1 fragment containing the miR-596 binding site or LETM1 3'-UTR was subcloned into the psiCHECK2 dual-luciferase vector (Promega). The luciferase reporter plasmid was co-transfected into EOR cells with miR-596-mimics or negative control. Relative luciferase activity was measured using a dual-luciferase reporter assay system (Promega) according to the manufacturer's instructions.

### CCK-8 and colony formation assay

For CCK-8 analysis, cells were seeded at a density of 1000 cells/well into 96 well plates, and 10 μl of CCK-8 (Dowjindo, Kyushu, Japan) was added to each well on days 0-5. The cells were then incubated at 37°C for 2 hours, and the optical density was measured at 450 nm. In the colony formation assay, 400 cells were seeded into 6-well plates and cultured at 37°C for 10 days. The colonies were then fixed with methanol and stained with hematoxylin.

### Wound healing assay and transwell assay

In the wound healing assay, cells were seeded in 6-well plates and cultured to a sub-fusion state. After starving for 24 hours in a serum-free medium, the monolayer cells were linearly scraped to introduce artificial wounds captured at 0 hours and 24/48 hours. For cross-well migration and intrusion testing, a cross-well chamber (Corning, USA) with a pore size of 8 μm was coated with or without matrix gel (BD Biosciences). Subsequently, 5 x 10^4^ or 1 x 10^5^ cells suspended in serum-free medium were seeded in the upper chamber, and medium supplemented with 10% FBS was placed in the lower chamber. After 12 or 24 hours of culture, the cells were fixed, stained, and counted using an inverted microscope.

### FISH

Nuclei and cytoplasmic RNA were separated by NE-PERTM nuclear extractant (Invitrogen) and analyzed by quantitative RT-PCR. For FISH testing, cells were seeded in 24 wells with glass coverslips for 24 hours. After immobilization and permeabilization, cells were hybridized with 20 μM Cy3-labeled RHPN1-AS1 or U6-FISH probe mix (Ribobio), and nuclei were stained with 6-diamino-2-phenylindole (DAPI). The image was observed with a confocal laser scanning microscope (Olympus FV1000, Japan).

### Animal experiment

Animal experiments were carried out in the Animal Experimental Center, Wenling Maternal and Child Health Care Hospital. Female BALB/c nude mice (4-5 weeks old) were obtained from the Beijing Vital River Laboratory Animal Technology Co, Ltd. (Beijing, China). 1×10^6^ ES-2-Scrambled or ES-2-shRHPN1-AS1 cells were injected subcutaneously on the right side of the mice. After 6 weeks, mice were sacrificed by intraperitoneal injection of 200 mg/kg pentobarbital, and tumors were weighed and measured. All animal experiments were performed following the Guide for the Care and Use of Laboratory Animals.

### In situ hybridization (ISH) and immunohistochemistry (IHC)

As previously described [10], ISH was performed using an ISH kit (Wuhan Bobst Bioengineering Co., Ltd., China) according to the manufacturer's instructions. For IHC assay, sections were incubated with rabbit anti-LETM1 monoclonal antibody (1:800, Abcam) overnight at 4°C with normal goat serum as a negative control.

### Statistical analysis

Statistical analysis was performed using SPSS 21.0 software. All data are displayed as mean ± standard deviation (SD). Student's t-test or x^2^ test analyzed comparisons. Survival was determined by the Kaplan-Meier method. The effect of variables on survival was determined by univariate and multivariate Cox proportional hazard models. P values <0.05 were considered statistically significant.

### Ethics approval

This research has been carried out following the World Medical Association Declaration of Helsinki and that all subjects provided written informed consent. The medical ethics committee approved this study of Wenling Maternal and Child Health Care Hospital.

## Supplementary Material

Supplementary Table 1
