# Supplementary material for: Long non-coding RNA RHPN1-AS1 promotes tumorigenesis and metastasis of ovarian cancer by acting as a ceRNA against miR-596 and upregulating LETM1
Source: Aging (Albany NY). 2020 Mar 12;12(5):4558–72. doi: 10.18632/aging.102911 (PMC7093190; doi:10.18632/aging.102911)
Supplement: Supplementary Table 1 [file aging-12-102911-s001..pdf]

SUPPLEMENTARY TABLE

Supplementary Table 1. Clinical characteristics of EOC patients according to the high and low expression of *RHPN1-AS1.*

Characteristics	*n*	Expression of *RHPN1-AS1*		*P*
		Low, *n* (%)	High, *n* (%)	
**Age (yrs)**				
≤45	36	19 (44.0)	17 (40.0)	0.684
>45	50	24 (56.0)	26 (60.0)	
**Gender**				
Male	66	29 (67.4)	37 (86.0)	**0.003***
Female	20	14 (32.6)	6 (14.0)	
**T Stage**				
T1-T2	12	4 (9.3)	8 (18.6)	0.237
T3-T4	74	39 (90.7)	35 (81.4)	
**N Stage**				
N0-N1	50	26(78.0)	24 (60.0)	0.581
N2-N3	36	17(22.0)	19(40.0)	
**TNM Stage**				
III	51	25 (58.1)	26 (60.4)	1.592
IV	35	18 (41.9)	17 (39.6)	
**Distant metastasis**				
Yes	15	3 (11.6)	12 (18.6)	**0.001***
No	71	40 (88.4)	31 (81.4)	
**Death**				
Yes	27	9 (20.9)	18(41.9)	**0.001***
No	59	34 (79.1)	25 (58.1)	

*indicate $P<0.05$. *P* value is determined by χ^2 and Fisher's exact tests.
